# High-Throughput Plant Phenotyping for Developing Novel Biostimulants: From Lab to Field or From Field to Lab?

**DOI:** 10.3389/fpls.2018.01197

**Published:** 2018-08-14

**Authors:** Youssef Rouphael, Lukáš Spíchal, Klára Panzarová, Raffaele Casa, Giuseppe Colla

**Affiliations:** ^1^Department of Agricultural Sciences, University of Naples Federico II, Portici, Italy; ^2^Department of Chemical Biology and Genetics, Centre of the Region Haná for Biotechnological and Agricultural Research, Faculty of Science, Palacký University, Olomouc, Czechia; ^3^AgroBioChem, s.r.o., Bystročice, Czechia; ^4^Photon Systems Instruments, spol. s.r.o., Drasov, Czechia; ^5^Department of Agricultural and Forestry Sciences, University of Tuscia, Viterbo, Italy

**Keywords:** bioassaying, functional characterization, high-throughput screening, imaging methods, integrative phenotyping, mode of action, morpho-physiological traits, nutrient use efficiency

## Abstract

Plant biostimulants which include bioactive substances (humic acids, protein hydrolysates and seaweed extracts) and microorganisms (mycorrhizal fungi and plant growth promoting rhizobacteria of strains belonging to the genera *Azospirillum*, *Azotobacter*, and *Rhizobium* spp.) are gaining prominence in agricultural systems because of their potential for improving nutrient use efficiency, tolerance to abiotic stressors, and crop quality. Highly accurate non-destructive phenotyping techniques have attracted the interest of scientists and the biostimulant industry as an efficient means for elucidating the mode of biostimulant activity. High-throughput phenotyping technologies successfully employed in plant breeding and precision agriculture, could prove extremely useful in unraveling biostimulant-mediated modulation of key quantitative traits and would also facilitate the screening process for development of effective biostimulant products in controlled environments and field conditions. This perspective article provides an innovative discussion on how small, medium, and large high-throughput phenotyping platforms can accelerate efforts for screening numerous biostimulants and understanding their mode of action thanks to pioneering sensor and image-based phenotyping techniques. Potentiality and constraints of small-, medium-, and large-scale screening platforms are also discussed. Finally, the perspective addresses two screening approaches, “lab to field” and “field to lab,” used, respectively, by small/medium and large companies for developing novel and effective second generation biostimulant products.

## Plant Biostimulants: What They Are and Their Effects on Morpho-Physiological Traits of Crops

**FIGURE 1:**
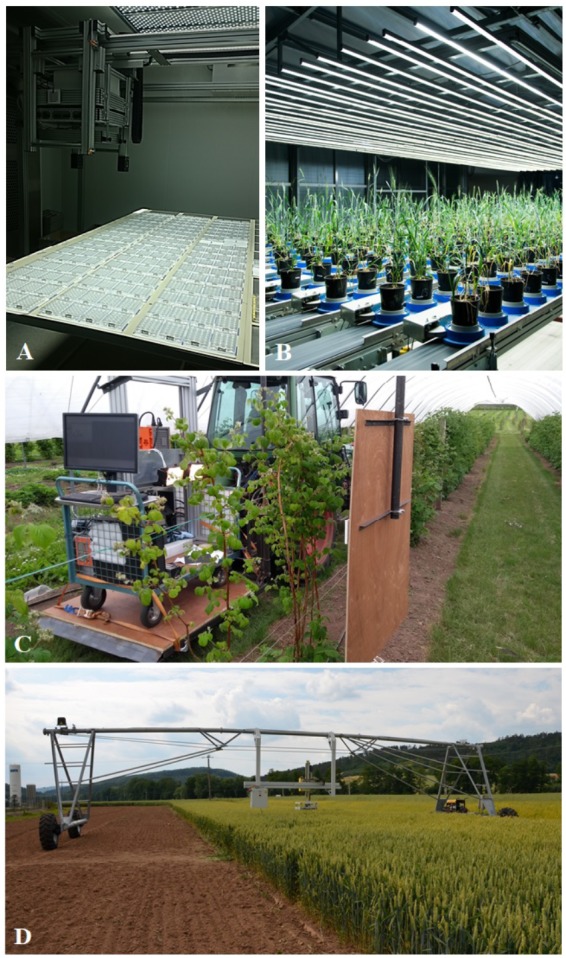
High-throughput plant phenotyping platforms: **(A)** small scale phenotyping platform consisting of XYZ PlantScreen^TM^ growth-chamber with automatic top view RGB imaging (Photon System Instruments, Czechia) for screening biostimulant substances based on the changes on *Arabidopsis* rosette growth in multi-well plates at Palacký University in Olomouc, Czechia; **(B)** medium-scale phenotyping platform PlantScreen^TM^ Modular System (Photon System Instruments, Czechia) with integrated high-resolution RGB, chlorophyll fluorescence, thermal and both VNIR and SWIR hyperspectral imagers for high-precision digital plant phenotyping and plant cultivation of mid-scale size up to large plants in greenhouse or semi-controlled environment; **(C)** Phenomobile for fruit trees and berry bushes developed at the James Hutton Institute (Scotland, United Kingdom), with VNIR and SWIR hyperspectral imagers (Williams et al., 2017; photo courtesy of H. G. Jones); **(D)** Large scale automated field phenotyping system. PlantScreen^TM^ Field System is autonomous mobile platform with multi-functional sensor platform mounted on an XZ-robotic arm with high-resolution visible, chlorophyll fluorescence, thermal infrared, hyperspectral imagers, and 3D laser sensor (Photon System Instruments, Czechia).

The term “biostimulant” was first introduced by Zhang and Schmidt (1997) in an online article of the Grounds Maintenance Journal describing them as “materials that, in minute quantities, promote plant growth." The biostimulants mentioned were humic acids and seaweed extracts, and their action on plants was proposed to be essentially hormonal. The term was subsequently adopted by many scientists to denote “substances and/or microorganisms applied to plants with the intention to enhance nutrition efficiency, abiotic stress tolerance and/or crop quality traits, regardless of its nutrients content” (du Jardin, 2015). From a regulatory point of view, there is no agreement worldwide defining plant biostimulants and many countries lack a legal framework. Within the EU, there is an ongoing revision of regulation aiming to establish a common legal framework for biostimulants, currently fragmented across Member States. Under the new regulation, plant biostimulants will be CE marked as fertilizing products stimulating plant nutrition processes independently of the products’ nutrient content with the sole aim of improving one or more of the following characteristics of the plant: nutrient use efficiency, tolerance to abiotic stress, and crop quality. Plant biostimulants are defined more by the plant response they elicit than by their makeup, since the category entails diverse substances and microorganisms such as humic acids, protein hydrolysates, seaweed extracts, silicon, mycorrhizal fungi, and nitrogen-fixing bacteria (Colla and Rouphael, 2015). Plant biostimulants can influence phenotypic traits and improve yield by enhancing crop stress-tolerance and nutrient uptake and assimilation. In most species, foliar or root application of plant biostimulants improves leaf pigmentation, photosynthetic efficiency, leaf number and area, shoot and root biomass, as well as fruit number and/or mean weight, especially under adverse environmental conditions (Ertani et al., 2013, 2014; Colla et al., 2015; Lucini et al., 2015, 2018; Rouphael et al., 2017). Precise and accurate assessment of phenotypic variables is critical for unraveling and quantifying the biostimulant activity of various products. High-throughput phenotyping technologies are receiving increasing attention for purposes of product screening and development as efficient means to (1) automated, non-destructive online monitoring of multiple morpho-physiological plant traits; (2) time-series measurements necessary for following the progression of growth, plant performance, and stress responses of individual plants at high-resolution; (3) reduced cost, labor, and time for analyses through automatization, remote sensing, improved data integration, and experimental design. High-throughput phenotyping technologies have been successfully employed in plant breeding (Araus and Cairns, 2014; Tardieu et al., 2017), however, their application in assessing plant biostimulant action has been limited (Petrozza et al., 2014). The current perspective article examines the potential benefits arising from the use of high-throughput phenotyping platforms (**Figure 1**) in biostimulant product screening and discusses current advances in plant phenotyping in the context of developing effective biostimulants.

## High-Throughput Phenotyping Platforms to Assess the Biostimulant Activity

### Small-Scale Screening Platforms

Screening platforms based on the semi-automated or automated bioassaying of plant/tissues traits using simple read-outs might be useful for identification of new biostimulants as well as for mode of action studies. Such platforms should allow parallel testing of large amounts of samples giving opportunity of high-throughput screening campaigns comparable to the chemical biology pipelines (Humplík et al., 2015). The advantage can lie in the possible miniaturization of the assays and use of simple and fast ways of biological response evaluation (De Diego et al., 2017). Further, because biostimulants represent various types of products including complex mixtures of biologically active compounds, testing should be done in broad concentration ranges offering evaluation of concentration-dependent effects. Importantly, the testing should cover analyses of the performance of a biostimulant in various stress conditions. This can be achieved mainly through bioassays in the platforms located in fully controlled environment allowing setting-up of various stress conditions such as temperature (heat/cold) and light (low/high intensity). The multivariate approach further counts with application of other stresses including low nutrients, salt, drought, or heavy metals. The higher level then represents cross-testing of a biostimulant in a broad concentration range against a concentration range of various stressors, or even their combinations. Such a highly complex screening approach can be highly efficient and lead to identification of novel biostimulants with various modes of action. Hence, the limiting factor of the screening platforms is the real throughput that depends on the level of automation, platform capacity, and the number of variants, which is in turn determined by the number of plants per variant and the number of technical replicates of each variant (De Diego et al., 2017). *Arabidopsis thaliana*, a classical model in plant biology, offers important advantages for phenotype-based high-throughput screening approaches. Bioassays using the *in vitro* grown *Arabidopsis* have high potential to be used in small-scale platforms for screening novel biostimulants applied through the growth medium. Several recently published protocols are based on RGB imaging of *Arabidopsis* shoot (rosette) growth. Miniaturization of the bioassay to the multi-well plates allows increasing the throughput to thousands of samples. *Arabidopsis* grown *in vitro* in 24-well plates were used for screening of growth regulator activity of a library of 10,000 compounds (Rodriguez-Furlán et al., 2016). Moreover, in this work the transferability of the results obtained with the model plant *Arabidopsis* to other crops of commercial interest, such as tomato, lettuce, carrots, has been also demonstrated (Rodriguez-Furlán et al., 2016). Recently, an automated method for high-throughput screening of *Arabidopsis* rosette growth in multi-well plates allowing measurement of 11,000 plants in less than 2 h has been presented by De Diego et al. (2017). In this method, several traits such as changes in the rosette leaf area, relative growth rate, survival rate and homogeneity of the population are scored using fully automated RGB imaging and subsequent image analysis. This method was successfully validated on example of multivariate analysis of rosette growth in different salt concentrations and the interaction with varying nutritional composition of the growth medium (De Diego et al., 2017). Many biostimulant products can directly or indirectly modify the plant hormone homeostasis of a treated plant. Principle of a facile forward chemical screening methodology for intact *Arabidopsis* seedlings harboring the β-glucuronidase (GUS) reporter under plant hormone-responsive promoters can be adapted for semi-automated testing in 96-well plates (Halder and Kombrink, 2015). Several existing transgenic *Arabidopsis* lines can be employed in such a lab-scale assay for multiple analyses of the effect of a biostimulant on the individual signaling pathways of cytokinins (ARR5::GUS), auxins (DR5::GUS), salicylic acid (PR1::GUS), abscisic acid (DC3::GUS), or bacterial elicitors such as flagellin (WRKY29::GUS). Such a complex assay could represent complementary tool for unraveling the mode of action of selected biostimulants. The potential pipeline of a biostimulant testing small-scale screening platform may consist of a sequence of automated assays determining the *Arabidopsis* performance under different growth conditions and the response to different abiotic stress treatments, followed by other species-based bioassays confirming applicability in crops. The next approach can be represented by complex phenotyping of selected variants combining various methods of automated, non-destructive, and simultaneous analyses of plant growth, morphology, and physiology in the medium-large screening platforms.

### Medium-Large Screening Platforms

Medium-large screening platforms are fully automated robotic systems usually installed in controlled environment or semi-controlled greenhouse conditions and are designed for automated cultivation, handling, and non-invasive monitoring of plants in throughput for a range of few up to several hundreds of plants. Plants can be dynamically monitored for many morpho-physiological traits related to growth, yield, and performance throughout their development or onset, progression, and recovery from abiotic stress. Biostimulant functional characterization in plants can be thus monitored in high-precision and high-resolution in a given phase of plant development and/or plant response to environmental conditions, depending on the target substance application or type of experimental layout. In terms of dimensions, phenotyping platforms are available for plants ranging from *Arabidopsis*, broadly used as a model plant also in biostimulant research field (Rodriguez-Furlán et al., 2016; De Diego et al., 2017), up to platforms providing technological solutions for screening complex morpho-physiological traits in mature crop plants such as barley, rice, soybean, or vegetable crops. Standard medium-to-large phenotyping platforms integrating one or multiple watering and weighing units ensure that a precise irrigation system with optional controlled nutrient delivery on plant specific basis can be used. This can be a key element for studies when biostimulant action is addressed together in combination with abiotic stress such as salinity or drought stress and/or when specific nutrient regime is applied, and nutrient use efficiency is studied throughout plant development. Integration of automated and programmable spraying unit into the phenotyping pipeline further extends the capacities of the platform by maximizing the standardization of the biostimulant application and/or availability for different modes of applications (e.g., drench vs. spraying). In general, many developmental processes can be actively regulated following biostimulant application. Multiple functions of biostimulant activity on plants can be characterized by growth-promoting features, enhancement of nutrition efficiency, and abiotic stress tolerance (du Jardin, 2015). This broad spectrum of traits can be quantitatively described and qualitatively differentiated by so-called integrative phenotyping in multi-sensoric phenotyping platforms including imaging sensors for visible imaging (RGB imaging) and/or 3D imaging, imaging spectroscopy (hyperspectral imaging), thermal infrared imaging, and chlorophyll fluorescence imaging. The integrative phenotyping approach based on integration of multiple read-outs from various imaging and non-imaging sensors available in these types of platforms (Humplík et al., 2015) allows to draw more complex images on the possible mode of action of a biostimulant under specific environmental conditions. Range of commercial phenotyping platforms is nowadays available with different specificities and key imaging sensor features. The reader is advised to view recent reviews with overview of available imaging sensors and commercial technologies on the market (Humplík et al., 2015; Rahaman et al., 2015; Mishra et al., 2016). The platforms can be either built within large controlled-environment chambers or implemented inside of greenhouse environments. Implementation of multiple imaging and non-imaging sensors (e.g., environmental sensors) within the phenotyping platforms provides the possibility to design species-specific phenotyping protocols in order to understand: plant growth dynamics and performance via RGB imaging; plant’s photosynthetic capacity and ability to harvest light energy by chlorophyll fluorescence imaging, stomatal conductance, and water transpiration rates of plants by measuring leaf and canopy temperature with thermal imaging sensors; biochemical composition of plants by quantification of spectral reflectance profiles with hyperspectral imaging, precise architecture, and shape of the plants by 3D imaging. Above all, standardized data management routines and sophisticated image analysis algorithms are implemented within the general phenotyping pipelines (Tardieu et al., 2017). Altogether by using advanced data analysis algorithms and statistical analysis for the multi-dimensional phenotype data that are resulting from integrative phenotyping approach, the broad spectrum of morpho-physiological traits can be clustered and the traits correlating with the given phase of biostimulant application or the stress response can be identified. The so far above-described phenotyping approaches can be successfully used for in-depth characterization of biostimulants action in a range of plants species, however, the read-out refers solely to above-ground morpho-physiological features. The below-ground features referring to root system architecture and its function are not analyzed as routinely as shoot features but certainly should not be neglected. Range of automated and semi-automated phenotyping platforms are currently available for quantitative and dynamic analysis of root growth and architecture (Paez-Garcia et al., 2015). However, in most cases, and especially for crop species of bigger size, range of technical limitations must still be overcome. Major challenges for root phenotyping remain in providing high throughput level tools with relevant growing conditions and with appropriate spatial and time resolution of image acquisition and this in both time and cost-effective manner.

### Field Phenotyping Systems

Many of the effects of biostimulants are related to improvements of the functioning of root systems and their interaction with the soil environment and to improved mechanisms of tolerance to environmental stresses (Calvo et al., 2014). Therefore, it is clear that controlled environments do not always provide a realistic context for their assessment. Soil characteristics, rainfall, temperature, and weather, along with the presence of diseases, insect pests and weeds, interact with the mechanisms of action of biostimulants, thereby influencing their efficacy across years. Additionally, crop physiological processes acting at the canopy scale, when plants are grown together in the field, have their own specific mechanisms, such as root mutual relationships and competitive effects that interact with those influenced by biostimulants in the single plant, when grown alone in a pot. In recent years there has been impressive progress in the development of approaches for open-field phenotyping (Araus and Cairns, 2014; Shakoor et al., 2017), and the accuracy of proximal or remote sensing systems for ground-based to aerial platforms is dramatically increasing. The use of such systems opens the way to a spectacular increase in the capability of screening large number of genotypes in the field, with non-destructive, repeated, objective observations, without the requirement of an extensive labor force. It is not only for plant breeding that these systems could be used, but also for physiological and agronomic studies, including the assessment of biostimulants. Sensors can be deployed on the ground, on fixed or mobile platforms, so that the distance to the target ranges from less than one to a few ten meters. Fixed platforms, in which the sensors do not move, include towers (Naito et al., 2017), tripods (Friedli et al., 2016), and wireless sensor networks (WSN) (Jones et al., 2018). Mobile ground platforms range from tractor-based systems (Enciso et al., 2017; Salas Fernandez et al., 2017), to manually driven buggies (Deery et al., 2014), or autonomous mobile rovers (Madec et al., 2017), to fixed rails (Virlet et al., 2017), or wires (Kirchgessner et al., 2017). Alternatively, phenotyping systems can be carried by unmanned aerial vehicles (UAV) (Sankaran et al., 2015; Yang et al., 2017) or blimps/balloons, in which case the distance from the target is generally of the order of 30–150 m, so they could be considered as remote sensing systems. There are advantages and disadvantages for each platform type, extensively discussed in previous reviews (Deery et al., 2014; Shakoor et al., 2017; Yang et al., 2017). In general, ground-based systems have a higher spatial resolution (i.e., ground sampling distance) and the possibility of assembling multiple-sensor arrays, combining, for example, hyperspectral, thermal, and lidar sensors. Conversely UAVs are limited by a small payload of just one or two instruments. On the other hand, ground platforms can be slow to move, so that environmental conditions may change by the time they move from one plot to another. This is a disturbing effect for some spectral (Virlet et al., 2017) and thermal sensing systems (Deery et al., 2014), which are sensitive to the effect of varying solar irradiance, for example, in the case of sky conditions with scattered clouds. Additionally, fixed ground-based systems constrain the possibility of changing the experimental plot area, sometimes preventing a sound agronomic practice of crop rotation. They also pose strong limitations to conventional soil preparation (i.e., tillage), because the platform only covers a fixed small land area, where conventional agricultural machinery cannot be used (Kirchgessner et al., 2017; Virlet et al., 2017). Thus, for biostimulants assays, mobile systems should be preferred. In general, the suitability of the platform will vary in relation to the objectives of the study and on the plant variables that need to be estimated, as well as on the accuracy required in their estimation. In the case of biostimulants, the variables of interest that can be monitored from current field phenotyping systems, are those related to canopy structure and growth, photosynthesis, water relations, and leaf biochemistry. These variables should be generally estimated with an accuracy better than 10%, in order to be able to discriminate the effect of biostimulants (Calvo et al., 2014). Ground-based lidar or terrestrial 3D laser scanning systems seem to provide the most accurate and versatile tool for canopy structure and functioning assessment (Deery et al., 2014; Kjaer and Ottosen, 2015; Friedli et al., 2016), better than for example, RGB structure from motion techniques (Madec et al., 2017). Infrared thermography, when due attention is paid to ancillary measurements and/or reference surfaces, allows the assessment of transpiration and stomatal functioning (Jones et al., 2018). Close-range imaging spectroscopy (Mishra et al., 2017) seems to be the most promising tool for the assessment of tissue biochemistry, though technical issues exist, related to the data acquisition configuration, for example, for line scanners and for conversion into absolute reflectance (Deery et al., 2014), as well as for heavy data processing (Virlet et al., 2017). For the assessment of photosynthetic functioning and stress responses, fluorescence imaging has great potential, despite technical limitations of some techniques in field conditions (e.g., illumination) (Shakoor et al., 2017; Virlet et al., 2017). The possibility to assess root structure and functioning is not available in current systems (Pauli et al., 2016) although it would be extremely interesting for biostimulant assessment. In this context, the mapping of soil properties, for example, by geoelectrical sensors or/and hyperspectral bare soil data (Casa et al., 2013), rather than root structure *per se* provides a potentially powerful ally to direct root detection.

## Biostimulant Development Process: From Lab to Field or From Field to Lab?

Biostimulant activity is modulated by interacting factors such as plant genotype, growing conditions, dose, and application time. Crops in open field are faced with multiple/combined abiotic stresses difficult to reproduce in controlled environment. Moreover, the performance of microbial biostimulants depends on native soil microflora, physical, and chemical conditions of the soil and climatic factors. For these reasons, biostimulants screened in controlled environment do not always perform as expected under field conditions. An effective approach would be to screen substances/microorganisms for biostimulant activity under real field conditions and then use small-medium phenotyping platforms in controlled-environment experiments to understand their mode of action on model plants like *Arabidopsis*. Although this approach seems most appropriate for identifying effective biostimulant products, many companies initiate the screening process in controlled environment to shorten the time needed to identify new bioactive substances and beneficial microorganisms and to narrow the number of products later tested in real field conditions. This “lab to field” approach is mostly used by SMEs to reduce the cost of field testing for product development. On the contrary, a “field to lab” approach is especially adopted by big companies using large-scale field testing to develop efficient biostimulants under real growing conditions. For instance, Albaugh, LLC, and Italpollina United States, Inc. recently announced a long-term strategic collaboration to deliver biological seed treatment solutions for boosting crop yields in a sustainable way. In 2015–2017, the Alliance tested more than 50 seed treatments (vegetal-based protein hydrolysates, *Rhizoglomus irregulare* BEG72, *Funelliformis mossae* BEG234, and *Trichoderma atroviride* MUCL 45632) across 330 field trials in more than 100 locations in United States (Bonini et al., 2017). The best performing products were also tested in trials under controlled environment to investigate their mode of action using a ‘multi-omics’ approach. This collaboration has resulted in the launch of several biological seed treatments (BIOST^®^VPH100; BIOST^®^Mycorrhizae 100; BIOST^®^Trichoderma 100) for growers of field crops such as canola, corn, cotton, rice, sorghum, soybeans, sugarbeets, and wheat^1^.

## Author Contributions

YR and GC had the original idea to write the perspective article and GC coordinated the manuscript preparation. YR and GC wrote the section “Plant Biostimulants: What They Are and Their Effects on Morpho-Physiological Traits of Crops” and “Biostimulant Development Process: From Lab to Field or From Field to Lab?” LS wrote the section “Small-Scale Screening Platforms.” KP wrote the section “Medium-Large Screening Platforms.” RC wrote the section “Field Phenotyping Systems.” All authors contributed significantly to improve the final version of the article.

## Conflict of Interest Statement

The authors declare that the research was conducted in the absence of any commercial or financial relationships that could be construed as a potential conflict of interest.

## Footnotes

1 http://www.albaughllc.com/north-america/seed-treatment/biost-platform/

## References

[B1] Araus J. L. Cairns J. E. (2014). Field high-throughput phenotyping: the new crop breeding frontier. *Trends Plant Sci.* 19 52–61. 10.1016/j.tplants.2013.09.008 24139902

[B2] Bonini P. Long D. H. Canaguier R. Colla G. Leman J. (2017). *Seed Treatments with Endophytic Fungi and Biostimulant Compounds Enhance Yield of Corn and Soybean. the 3rd World Congress on the Use of Biostimulants in Agriculture, 27-30 November 2017*. Miami, FL: Book of Abstracts, 147.

[B3] Calvo P. Nelson L. Kloepper J. W. (2014). Agricultural uses of plant biostimulants. *Plant Soil* 383 3–41. 10.1007/s11104-014-2131-8

[B4] Casa R. Castaldi F. Pascucci S. Basso B. Pignatti S. (2013). Geophysical and hyperspectral data fusion techniques for in-field estimation of soil properties. *Vadose Zone J.* 12:4 10.2136/vzj2012.0201

[B5] Colla G. Rouphael Y. (2015). Biostimulants in horticulture. *Sci. Hortic.* 196 39-48. 10.1016/j.scienta.2015.10.044

[B6] Colla G. Rouphael Y. Bonini P. Cardarelli M. (2015). Coating seeds with endophytic fungi enhances growth, uptake nutrient, yield and grain quality of winter wheat. *Int. J. Plant Prod.* 9 171–190.

[B7] De Diego N. Fürst T. Humplík J. F. Ugena L. Podlešáková K. Spíchal L. (2017). An automated method for high-throughput screening of Arabidopsis rosette growth in multi-well plates and Its validation in stress conditions. *Front. Plant Sci.* 8:1702. 10.3389/fpls.2017.01702 29046681 PMC5632805

[B8] Deery D. Jimenez-Berni J. Jones H. Sirault X. Furbank R. (2014). Proximal remote sensing buggies and potential applications for field-based phenotyping. *Agronomy* 4 349–379. 10.3390/agronomy4030349

[B9] du Jardin P. (2015). Plant biostimulants: definition, concept, main categories and regulation. *Sci. Hortic.* 196 3–14. 10.1016/j.scienta.2015.09.021

[B10] Enciso J. Maeda M. Landivar J. Jung J. Chang A. (2017). A ground based platform for high throughput phenotyping. *Comp. Elect. Agric.* 141 286–291. 10.1016/j.compag.2017.08.006

[B11] Ertani A. Pizzeghello D. Francioso O. Sambo P. Sanchez-Cortes S. Nardi S. (2014). *Capsicum chinensis* L. growth and nutraceutical properties are enhanced by biostimulants in a long-term period: chemical and metabolomic approaches. *Front. Plant Sci.* 5:375. 10.3389/fpls.2014.00375 25136346 PMC4117981

[B12] Ertani A. Schiavon M. Muscolo A. Nardi S. (2013). Alfalfa plant-derived biostimulant stimulate short-term growth of salt stressed Zea mays L. plants. *Plant Soil* 364 145–158.

[B13] Friedli M. Kirchgessner N. Grieder C. Liebisch F. Mannale M. Walter A. (2016). Terrestrial 3D laser scanning to track the increase in canopy height of both monocot and dicot crop species under field conditions. *Plant Methods* 12:9. 10.1186/s13007-016-0109-7 26834822 PMC4731982

[B14] Halder V. Kombrink E. (2015). Facile high-throughput forward chemical genetic screening by in situ monitoring of glucuronidase-based reporter gene expression in Arabidopsis thaliana. *Front. Plant Sci.* 6:13. 10.3389/fpls.2015.00013 25688251 PMC4310277

[B15] Humplík J. F. Lazár D. Husičková A. Spíchal L. (2015). Automated phenotyping of plant shoots using imaging methods for analysis of plant stress responses – a review. *Plant Methods* 11:29. 10.1186/s13007-015-0072-8 25904970 PMC4406171

[B16] Jones H. G. Hutchinson P. A. May T. Jamali H. Deery D. M. (2018). A practical method using a network of fixed infrared sensors for estimating crop canopy conductance and evaporation rate. *Biosyst. Eng.* 165 59–69. 10.1016/j.biosystemseng.2017.09.012

[B17] Kirchgessner N. Liebisch F. Yu K. Pfeifer J. Friedli M. Hund A. (2017). The ETH field phenotyping platform FIP: a cable-suspended multi-sensor system. *Funct. Plant Biol.* 44 154–168. 10.1071/FP16165 32480554

[B18] Kjaer K. H. Ottosen C. O. (2015). 3D laser triangulation for plant phenotyping in challenging environments. *Sensors* 15 13533–13547. 10.3390/s150613533 26066990 PMC4507705

[B19] Lucini L. Rouphael Y. Cardarelli M. Bonini P. Baffi C. Colla G. (2018). A vegetal biopolymer-based biostimulant promoted root growth in melon while triggering brassinosteroids and stress-related compounds. *Front. Plant Sci* 9:472. 10.3389/fpls.2018.00472 29692795 PMC5902679

[B20] Lucini L. Rouphael Y. Cardarelli M. Canaguier R. Kumar P. Colla G. (2015). The effect of a plant-derived protein hydrolysate on metabolic profiling and crop performance of lettuce grown under saline conditions. *Sci. Hortic.* 182 124–133. 10.1016/j.scienta.2014.11.022

[B21] Madec S. Baret F. De Solan B. Thomas S. Dutartre D. Jezequel S. (2017). High-throughput phenotyping of plant height: comparing unmanned aerial vehicles and ground lidar estimates. *Front. Plant Sci.* 8:2002. 10.3389/fpls.2017.02002 29230229 PMC5711830

[B22] Mishra K. B. Mishra A. Klem K. Govindjee (2016). Plant phenotyping: a perspective. *Ind. J. Plant Physiol.* 21 514–527. 10.1007/s40502-016-0271-y

[B23] Mishra P. Asaari M. S. M. Herrero-Langreo A. Lohumi S. Diezma B. Scheunders P. (2017). Close range hyperspectral imaging of plants: a review. *Biosyst. Eng.* 164 49–67. 10.1016/j.biosystemseng.2017.09.009

[B24] Naito H. Ogawa S. Valencia M. O. Mohri H. Urano Y. Hosoi F. (2017). Estimating rice yield related traits and quantitative trait loci analysis under different nitrogen treatments using a simple tower-based field phenotyping system with modified single-lens reflex cameras. *ISPRS J. Photog. Remote Sens.* 125 50–62. 10.1016/j.isprsjprs.2017.01.010

[B25] Paez-Garcia A. Motes C. M. Scheible W. R. Chen R. Blancaflor E. B. Monteros M. J. (2015). Root traits and phenotyping strategies for plant improvement. *Plants* 4 334–355. 10.3390/plants4020334 27135332 PMC4844329

[B26] Pauli D. Chapman S. C. Bart R. Topp C. N. Lawrence-Dill C. J. Poland J. (2016). The quest for understanding phenotypic variation via integrated approaches in the field environment. *Plant Physiol.* 172 622–634. 10.1016/j.isprsjprs.2017.01.010 27482076 PMC5047081

[B27] Petrozza A. Santaniello A. Summerer S. Di Tommaso G. Di Tommaso D. Paparelli E. (2014). Physiological responses to Megafol treatments in tomato plants under drought stress: a phenomic and molecular approach. *Sci. Hortic.* 174 185–192. 10.1016/j.scienta.2014.05.023

[B28] Rahaman M. M. Chen D. Gillani Z. Klukas C. Chen M. (2015). Advanced phenotyping and phenotype data analysis for the study of plant growth and development. *Front. Plant Sci.* 6:619. 10.3389/fpls.2015.00619 26322060 PMC4530591

[B29] Rodriguez-Furlán C. Miranda G. Reggiardo M. Hicks G. R. Norambuena L. (2016). High throughput selection of novel plant growth regulators: assessing the translatability of small bioactive molecules from Arabidopsis to crops. *Plant Sci.* 245 50–60. 10.1016/j.plantsci.2016.01.001 26940491

[B30] Rouphael Y. Colla G. Giordano M. El-Nakhel C. Kyriacou M. C. De Pascale S. (2017). Foliar applications of a legume-derived protein hydrolysate elicit dose dependent increases of growth, leaf mineral composition, yield and fruit quality in two greenhouse tomato cultivars. *Sci. Hortic.* 226 353–360. 10.1016/j.scienta.2017.09.007

[B31] Salas Fernandez M. G. Bao Y. Tang L. Schnable P. S. (2017). A high-throughput, field-based phenotyping technology for tall biomass crops. *Plant Physiol.* 174 2008–2022. 10.1104/pp.17.00707 28620124 PMC5543940

[B32] Sankaran S. Khot L. R. Espinoza C. Z. Jarolmasjed S. Sathuvalli V. R. Vandemark G. J. (2015). Low-altitude, high-resolution aerial imaging systems for row and field crop phenotyping: a review. *Eur. J. Agron.* 70 112–123. 10.1016/j.eja.2015.07.004

[B33] Shakoor N. Lee S. Mockler T. C. (2017). High throughput phenotyping to accelerate crop breeding and monitoring of diseases in the field. *Curr. Opin. Plant Biol.* 38 184–192. 10.1016/j.pbi.2017.05.006 28738313

[B34] Tardieu F. Cabrera-Bosquet L. Pridmore T. Bennett M. (2017). Plant phenomics, from sensors to knowledge. *Curr. Biol.* 27 R770–R783. 10.1016/j.cub.2017.05.055 28787611

[B35] Virlet N. Sabermanesh K. Sadeghi-Tehran P. Hawkesford M. J. (2017). Field scanalyzer: an automated robotic field phenotyping platform for detailed crop monitoring. *Funct. Plant Biol.* 44 143–153. 10.1071/fp16163 32480553

[B36] Williams D. Britten A. McCallum S. Jones H. G. Aitkenhead M. Karley A. (2017). A method for automatic segmentation and splitting of hyperspectral images of raspberry plants collected in field conditions. *Plant Methods* 13:74. 10.1186/s13007-017-0226-y 29118819 PMC5664591

[B37] Yang G. Liu J. Zhao C. Li Z. Huang Y. Yu H. (2017). Unmanned aerial vehicle remote sensing for field-based crop phenotyping: current status and perspectives. *Front. Plant Sci.* 8:1111. 10.3389/fpls.2017.01111 28713402 PMC5492853

[B38] Zhang X. Schmidt R. E. (1997). The impact of growth regulators on alpha-tocopherol status of water-stressed *Poa pratensis* L. *Int. Turfgrass Soc. Res. J.* 8 1364–2137.

